# A machine learning approach towards endometriosis screening using infrared spectra of urine

**DOI:** 10.1016/j.clinsp.2025.100760

**Published:** 2025-09-06

**Authors:** Matthews Silva Martins, Gabriela Barros Valente, Yasmin do Nascimento Pedra, Thaís Campos Ribeiro, Neide Aparecida Tosato Boldrini, Mara Rejane Barroso Barcelos, Francis L. Martin, Karin Kneipp Costa Rossi, Valerio Garrone Barauna

**Affiliations:** a Department of Physiological Sciences, Universidade Federal do Espírito Santo, Vitória, ES, Brazil; b Hospital Universitário Cassiano Antonio Moraes, Universidade Federal do Espírito Santo, Vitória, ES, Brazil; c Biocel UK Ltd, Hull, UK; d Department of Cellular Pathology, Blackpool Teaching Hospitals NHS Foundation Trust, Whinney Heys Road, Blackpool, UK

**Keywords:** Endometriosis, Urine, Screening, Machine learning, Spectroscopy

## Abstract

**Background:**

Endometriosis diagnosis is challenging due to non-specific symptoms that overlap with other gynaecological conditions. This study proposes a non-invasive Machine Learning (ML) ‒ based urine test using Attenuated Total Reflection Fourier Transform Infrared (ATR-FTIR) spectroscopy for rapid, high-throughput screening.

**Methods:**

A total of 302 symptomatic patients presenting with pelvic pain and MRI referral indications were recruited. After applying exclusion criteria, 100 patients (50 endometriosis-positive, 50 endometriosis-negative with other gynaecological conditions) were included. Urine samples were self-collected during the first visit and analysed via ATR-FTIR spectroscopy. Two Machine Learning (ML) algorithms, sensitivity-tuned and specificity-tuned, were developed using ∼1,700 spectral variables per patient to prioritize either sensitivity or specificity.

**Results:**

There were no statistically significant differences in patient characteristics between groups, as patients with negative results for endometriosis presented with other gynaecological disorders. The sensitivity-tuned algorithm achieved 93 % sensitivity and 57 % specificity, while the specificity-tuned version reached 93 % specificity but only 27 % sensitivity. Given an endometriosis prevalence of 30 % in symptomatic population, the sensitivity-tuned test reduced unnecessary MRI referrals by 42 %, prioritizing patients most likely to have endometriosis. The analysis time was 40 s per replicate, enabling same-day results.

**Conclusion:**

This proof-of-concept study demonstrates the clinical potential of a rapid, urine-based ML test to reduce diagnostic delays and imaging costs. Validation in larger, multi-center cohorts is underway to enhance robustness and generalizability.

## Highlights

• ML-based urine test enables screening for endometriosis (93 % sensitivity).
• 42 % reduction in unnecessary MRI referrals during endometriosis evaluation.
• Same-day results support early triage of symptomatic gynecologic patients.
• ATR-FTIR spectra combined with ML distinguish endometriosis biochemical profiles.
• Decision-support tool for stratifying symptomatic patients before imaging.

## Introduction

Endometriosis is a chronic, debilitating gynaecological disorder characterized by the presence of endometrial-like tissue (stroma and glands) outside the uterus. It affects approximately 10 % of women of reproductive age and impacts around 190 million women worldwide.^1^ The multi-systemic nature of the disease, along with its non-specific symptoms such as chronic pelvic pain, dysmenorrhea, and dyspareunia-poses significant challenges to early diagnosis.^2^

The gold standard for diagnosing endometriosis remains surgical visualization through laparoscopy, an invasive and costly procedure. Alternatively, increasing evidence supports the use of imaging techniques, particularly Magnetic Resonance Imaging (MRI), which can accurately detect and characterize endometrial lesions in various locations.^3^, ^4^, ^5^ However, while MRI is minimally invasive, it is also costly and often involves delays for patients in scheduling the exam and receiving results. Particularly in developing countries, and even with a public healthcare system (SUS, Sistema Único de Saúde) that is free to the entire population, such as Brazil, the waiting list for an MRI can be as long as 6‒12 months. Complementary approaches for endometriosis screening were also proposed, including the use of multiple biomarkers^6^, ^7^, ^8^ and symptom-based tools.^9^^,^^10^ However, none have met the accuracy criteria for a practical triage diagnostic test.

Consequently, there is a pressing need for reliable screening methods in clinical practice, as women with endometriosis currently face an average diagnostic delay of 7‒10 years.^11^ Pre-diagnosis endometriosis-related costs average $3,553 per patient, including ambulatory emergency visits and inpatient hospitalizations.^12^ Additionally, delays in diagnosis significantly reduce women's quality of life, leading to symptoms of anxiety, emotional distress, and depression.^13^ Infertility is also a common outcome, affecting 30 % to 50 % of patients with endometriosis. It can negatively impact patients' psychological well-being, generating feelings of inadequacy.^14^

The authors propose a non-invasive, rapid urine test to support clinical decision-making. The test uses Infrared (IR) light (*via* ATR-FTIR spectroscopy) to analyse urine samples, generating specific biochemical fingerprints for patients with and without the disease.^15^ Two Machine Learning (ML) algorithms were designed to learn disease patterns within these spectral signatures and classify new urine samples as positive or negative. Our test analyzes samples in 40 s per replicate, enabling same-day results, a critical advance for high-throughput screening.

Several new methods have been proposed to address the challenge of diagnosing endometriosis. Our primary goal was to develop machine learning algorithms to predict the likelihood of endometriosis based on the spectroscopy data of a urine sample. A positive result from our test would indicate an increased risk for endometriosis, making the patient a priority for further exams. Conversely, a negative result would suggest a lower risk, allowing the patient to continue routine follow-up based on clinical judgment. This protocol reduced unnecessary MRI scans, prioritized patients for expensive and invasive exams, and identified the best candidates for surgical intervention.

## Materials and methods

### Patient recruitment

The study was conducted at the gynaecology outpatient clinic of the Cassiano Antonio Moraes University Hospital, affiliated with the Federal University of Espírito Santo, Brazil. Ethical approval was obtained from the hospital's ethics committee (CAAE: 60880122.8.0000.5071), along with patient consent. This prospective, single-centre study is registered on ClinicalTrials.gov under the identifier NCT06426420 and was conducted in accordance with the STARD guidelines.

The authors recruited 302 symptomatic women aged 18‒55 years presenting with chronic pelvic pain and a clinical indication for pelvic MRI between August 2022 and July 2024. Exclusion criteria included pregnancy, inability to collect urine samples, incomplete medical history, and the absence of an MRI referral. Patients were approached during routine consultations without disrupting the flow of care. After providing informed consent, a questionnaire was completed for each patient. Additionally, they were given a universal urine collection container and instructed to collect a midstream urine sample. The urine was immediately aliquoted and stored at −20 °C.

All participant data were anonymised immediately after collection by removing personal identifiers (e.g., names and addresses) and replacing them with unique numerical codes. The anonymised datasets were stored on password-protected, encrypted servers accessible only to authorized study personnel. Urine samples were labelled using these codes, ensuring that analysts remained blinded to patient identities throughout the study. This protocol aligns with the Brazilian General Data Protection Law (LGPD).

Positive and negative cases of endometriosis were determined by an expert gynaecologist using MRI findings, gynaecological examinations, clinical symptoms, and patient history. Throughout the analysis process, each patient's diagnosis remained blinded to all analysts. This information was only disclosed during the calculation of diagnostic metrics.

### Participant characteristics

Data from the questionnaire, including symptoms, medical history, and physical examination, were analysed. Differences between groups (positive and negative for endometriosis) were assessed using the Chi-Square test or Fisher's exact test for qualitative variables and the *t*-test or Mann-Whitney test for quantitative variables, depending on whether the data followed a parametric or nonparametric distribution, respectively. A 95 % Confidence Interval was applied, and differences were considered statistically significant when *p* < 0.05.

### Sample analysis

Patient urine samples were analysed using an ATR-FTIR spectrometer (ALPHA II, Bruker) as previously published by our group.^16^, ^17^, ^18^ The technique is based on the interaction of IR light with the molecules present in the urine samples. The resulting interaction produces a specific biological signal that represents the complete biochemical profile of the samples (a data matrix containing ∼1,700 pieces of information), which can subsequently be used for classification by machine learning algorithms. For further details, readers are referred to the following articles.^15^^,^^19^^,^^20^

Before analysis, urine samples were thawed for up to 15 min at a controlled room temperature (20–24 °C). Each sample was vortexed, and 5 uL was directly pipetted in triplicate onto the spectrometer's crystal surface. Spectra were recorded using 32 co-added scans at a resolution of 4 cm^−1^. The analysis time for each replicate was approximately 40 s. The individual raw spectral dataset is presented in the Supplementary File (Figure S1).

### Algorithm training and disease prediction

A machine learning algorithm was designed to predict endometriosis based on the biological signal from the spectra. The algorithm was developed using 70 % of the data from 100 recruited patients, with an even distribution of positive and negative cases.

First, the averaged spectra of each patient were pre-processed to reduce noise, minimize the influence of water, and enhance the signal from informative variables (Fig. S2).^21^^,^^22^ The samples were then randomly split into training (70 %) and test (30 %) subsets. The training set was used to construct the classification model, while the test set was used to validate the model's predictive performance using diagnostic metrics.^23^ As the test set must consist of samples entirely unseen by the model (external validation), it must be kept completely isolated until the end of the training process.

The algorithm employed for the training process was a linear discriminant analysis combined with a genetic algorithm and Monte Carlo resampling (MC-GA-LDA). It performs multiple variable selection over a predefined number of iterations, resampling the spectra each time. By the end of the process, 100 classification models, each constructed with a different subset of variables, were generated using the training set.

The final prediction for the test set was determined by a consensus among the 100 models constructed using the three replicates of each patient. If two or more spectra were predicted as positive, the patient was classified as positive for endometriosis. If two or more spectra were predicted as negative, the patient was classified as negative for endometriosis. The performance metrics, including specificity, sensitivity, accuracy, Positive Predictive Value (PPV), Negative Predictive Value (NPV), F1-score, and area under the ROC Curve (AUC), are reported in the Results section based on the contingency matrix generated exclusively from the predictions on the test set.

## Results

### Recruitment overview

**Fig. 1:**
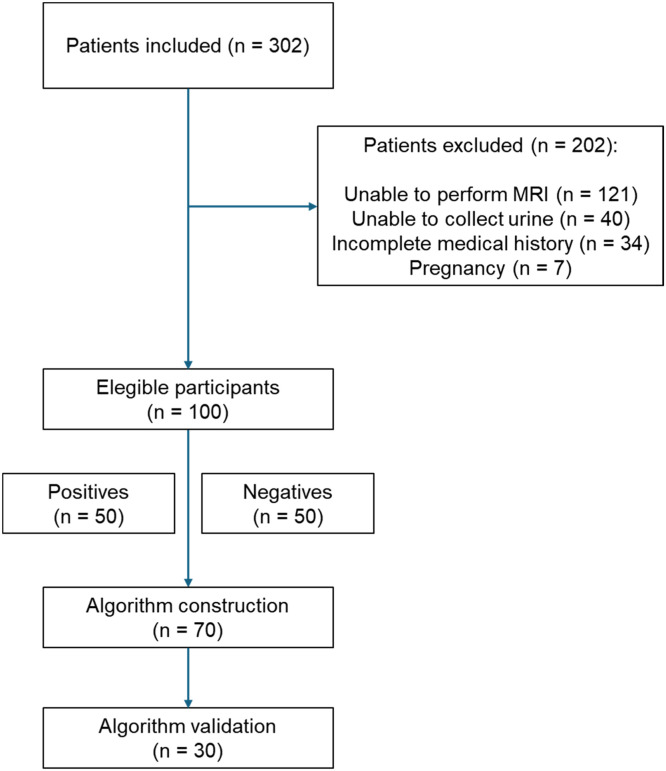
Flow of participants through recruitment for the study.

Of the 302 initially enrolled patients, 202 were excluded, predominantly due to an inability to undergo MRI (60 %), failure to collect urine (20 %), incomplete medical history (17 %), and pregnancy (3 %). The final number of patients eligible for the development of the ML-based test was 100 (Fig. 1).

### Patients’ characteristics

Data from the physical examination and the questionnaire, including symptoms and clinical history, are summarized in Table 1. No significant differences were observed between groups regarding age or any of the symptoms analysed. However, as expected, a significant difference was noted in the clinical history for the variable “Other gynaecological diseases”, with the negative group showing a higher proportion of patients with conditions other than endometriosis 44 % (*p* < 0.01).

**Table 1:** Demographic characteristics of the patients.

Characteristic	Negatives (*n* = 50)	Positives (*n* = 50)	p-value
Age (mean ± SD)	42 ± 8.1	39 ± 7.9	0.117
Painful periods			
Yes	46 (92 %)	45 (90 %)	0.727
No	4 (8 %)	5 (10 %)	
Pain during intercourse			
Yes	33 (66 %)	37 (74 %)	0.383
No	17 (34 %)	13 (26 %)	
Pain/burning during urination			
Yes	16 (32 %)	15 (30 %)	0.829
No	34 (68 %)	35 (70 %)	
Painful bowel movements			
Yes	19 (38 %)	21 (42 %)	0.683
No	31 (62 %)	29 (58 %)	
Infertility			
Yes	6 (22 %)	6 (22 %)	>0.99
No	44 (88 %)	44 (88 %)	
Other gynaecological diseases			
Yes	22 (44 %)	6 (12 %)	**<0.01**
No	28 (56 %)	44 (88 %)	
Family history of endometriosis			
Yes	13 (26 %)	8 (16 %)	0.220
No	37 (74 %)	42 (84 %)	
Pain on bimanual examination			
Yes	38 (76 %)	37 (74 %)	0.817
No	12 (24 %)	13 (26 %)	
Palpable masses on bimanual examination			
Yes	3 (6 %)	10 (20 %)	**0.037**
No	47 (94 %)	40 (80 %)	
Cervix injury			
Yes	2 (4 %)	6 (12 %)	0.140
No	48 (96 %)	44 (88 %)	

Regarding physical examination data, a significant difference was observed only in the variable “Palpable masses on bimanual examination”, with the positive group showing a higher proportion compared to the negative group (20 % vs. 6 %; *p* < 0.05).

### Algorithm construction

**Fig. 2:**
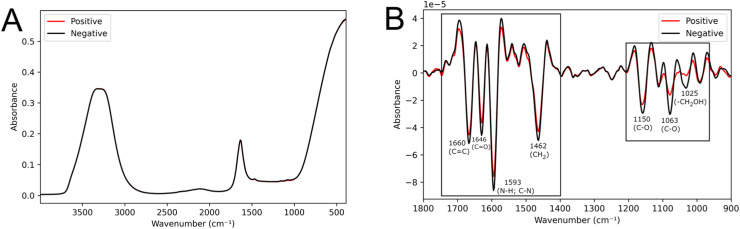
Averaged spectra from the positive and negative groups using raw data (A) and pre-processed data (B).

70 % of the samples (the training set) were utilized for constructing the MC-GA-LDA algorithm. Representative mean spectra from positive and negative groups are shown in Fig. 2A. In the pre-processed spectra (Fig. 2B), visual differences between groups were observed in the regions of 1700‒1550 cm^−1^, related to lipids (CH_2_ vibrations at 1462 cm^−1^ and *C* = *C* at 1660 cm^−1^) and proteins (Amide II at 1593 cm^−1^ and Amide I at 1646 cm^−1^); as well as in the regions 1200‒1000 cm^−1^, which are related to carbohydrates (C—O stretching vibration at 1150 cm^−1^, C—O stretching of ribose at 1063 cm^−1^ and vibrational frequency of -CH_2_OH groups of glucose, fructose, and glycogen at 1025 cm^−1^).^15^^,^^24^

The application of the MC-GA-LDA algorithm resulted in 100 distinct classification models. The variables (specific wavenumbers) selected by the algorithm that contributed the most to the correct classification are provided in Figure S3. The wavenumbers with the highest frequency of selection by the models correspond to the visual differences observed in the averaged spectra from the positive and negative groups (Fig. 2B).

**Fig. 3:**
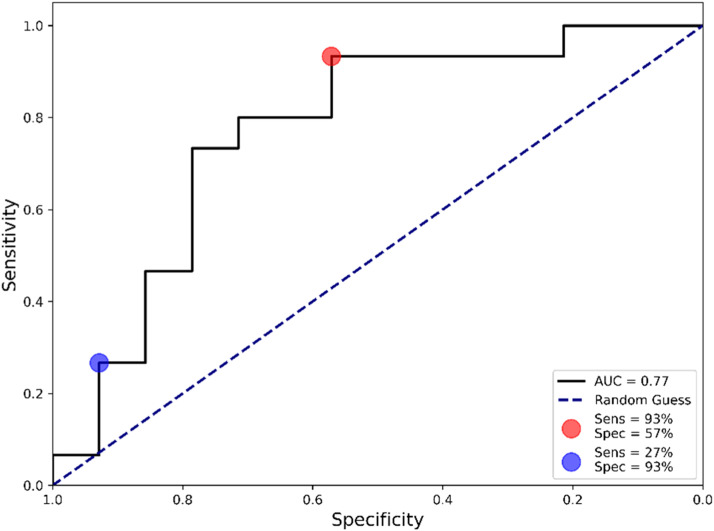
Receiver operating characteristic (ROC) curve showing the sensitivity-tuned (red circle) and specificity-tuned (blue circle) algorithms. Sens, Sensitivity; Spec, Specificity; AUC, Area Under the Curve.

The constructed algorithm reached a sensitivity of 73 %, a specificity of 79 %, an accuracy of 76 % and an F1-score of 0.76. The area under the ROC curve (AUC) is another useful metric for evaluating the validity of a diagnostic test.^25^ The reported AUC value was 0.77 (Fig. 3), indicating that the test has good discriminative ability. Depending on the diagnostic pathway or healthcare system requirements, the sensitivity and specificity of the test can be adjusted to maximize one over the other, which are referred to as sensitivity-tuned and specificity-tuned tests.

### Algorithm validation

Next, the sensitivity and specificity-tuned algorithms were validated in the remaining 30 % of samples not used in the previous step. When maximizing the sensitivity (Fig. 3, red circle), the test achieved a sensitivity of 93 % with a specificity of 57 %. Conversely, when maximizing the specificity (Fig. 3, blue circle), the test reached a specificity of 93 % and a sensitivity of 27 %. The complete diagnostic performance for both tuned algorithms is presented in Table 2.

**Table 2:** Diagnostic performance for both sensitivity-tuned and specificity-tuned algorithms.

	Sensitivity-tuned	Specificity-tuned
**Sensitivity**	93 %	27 %
**Specificity**	57 %	93 %
**PPV**	70 %	80 %
**NPV**	89 %	54 %
**F1-score**	80 %	40 %

PPV, Positive Predictive Value; NPV, Negative Predictive Value. The metrics are reported for the test set, which was equally distributed between positives and negatives.

## Discussion

Here, the authors have presented the interim analysis of our rapid and cost-effective ML-based urine test for detecting endometriosis and supporting clinical decision-making. The sensitivity-tuned algorithm reached a sensitivity of 93 % and a specificity of 57 %. Using urine eliminates the need for blood or tissue sampling, enabling a simple, painless, and patient-friendly collection process. The use of a non-invasive biofluid, along with the rapid 40-second analysis per replicate, represents the main strengths of this approach. By streamlining the diagnostic process, our test has the potential to shorten the time to diagnosis, minimize unnecessary examinations, and optimize healthcare resources.

**Fig. 4:**
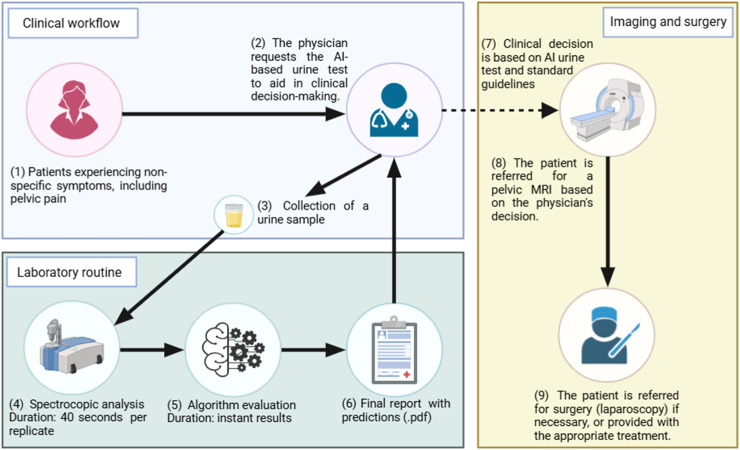
The proposed pathway for detecting endometriosis in patients with non-specific symptoms, using the machine learning-based urine test to assist in clinical decision-making. Created with BioRender.com.

Therefore, the authors propose a clinical workflow for detecting endometriosis using the ML-based urine test (Fig. 4).

Patients with non-specific symptoms would attend a standard consultation at secondary care facilities. The physician could request the ML-based urine test alongside other relevant biochemical exams during the consultation before considering an MRI referral. Urine samples would be self-collected on-site, and spectral data acquisition would take only 40 s, using 5 μL with no reagent or analytical pre-processing required. The algorithm would then analyse the spectral data in real-time, generating a final report with predictions, which would be sent to the physician alongside other examination results. The physician could then incorporate the test results into their clinical decision-making process, following standard guidelines.

Those patients with a positive result in the ML-based urine test could be prioritized for MRI examination within 1–2 weeks, provided their clinical presentation, symptoms, and physical examination findings are consistent with endometriosis. Conversely, patients with a negative result would follow the standard clinical workflow, depending on their overall assessment.

Assuming a symptomatic population of 1,000 patients with a 30 % prevalence of endometriosis, the sensitivity-tuned algorithm would identify 580 patients as candidates for pelvic MRI. Compared to the conventional approach, where all 1,000 patients would be referred for MRI (including 700 true negatives and 300 true positives), this represents a 42 % reduction in imaging referrals. While prioritizing sensitivity increases the number of false positives (301 patients incorrectly classified as positive), this is still significantly lower than the 700 negative patients who would undergo unnecessary MRI under the current standard care. Conversely, the algorithm produces only 21 false negatives. These patients may still be prioritized for MRI based on clinical reassessment, and if not, they can undergo the ML-based urine test in subsequent consultations.

Patients in countries with a public healthcare system, such as Brazil, can wait 6 to 12 months for a scan. By using the ML-based test, not only would the waiting list be reduced, but patients would also gain faster access to imaging and timely treatment initiation. Additionally, imaging expenditures for the healthcare system could be significantly minimized, freeing up resources for other areas. In Brazil, a pelvic MRI costs approximately R$900. A 42 % reduction in scans would save approximately R$378,000 for every 1,000 patients. This estimate accounts solely for imaging expenses; further savings could be realized from fewer follow-up appointments and complementary tests.

The authors present here a test that may serve as a first-line screening tool to prioritize patients with a higher likelihood of endometriosis for further evaluation, thereby reducing unnecessary examinations. Table 3 presents a comparison between current clinically validated diagnostic methods for endometriosis.

**Table 3:** Comparison of current diagnostic methods for endometriosis.

Method	Sensitivity	Specificity	Cost	Reference
Laparoscopy	98 %	87 %	R$ 5,000 ‒ R$ 15,000	^26^^,^^27^
MRI	94 %	77 %	R$ 700 ‒ R$ 900	^26^^,^^28^
TVS	79 %	94 %	R$ 200 ‒ R$ 400	^26^^,^^29^
Salivary miRNA test	97 %	94 %	R$ 5,100	30, 31, 32

MRI, Magnetic Resonance Imaging; TVS, Transvaginal Ultrasound; The metrics for laparoscopy are related to its use alone; The metrics for MRI and TVS are independent of the type and location of lesions.

Among them, the study by Bendifallah et al.^32^ is notable for also using ML tools for endometriosis screening, though based on the miRNA profile in saliva. The group proposes to validate a panel consisting of 109 previously sequenced miRNAs selected from 2,561 candidates in 200 patients. When validated in an additional cohort of 200 patients from five different medical centers, the test achieved a sensitivity of 96.2 % and a specificity of 95.1 %. The test is already commercially available in Europe through the French startup Ziwig.

The same group also attempts to apply ML algorithms to predict the likelihood of endometriosis using questionary data from patients. The authors selected 16 clinical and patient-reported symptom features from a pool of 500 features in 1,734 patients. Different algorithms were trained, and the best one achieved a sensitivity of 93 % and a specificity of 92 %.^33^ Existing reviews of symptom-based tools for detecting endometriosis highlight their limited clinical utility due to imbalanced performance metrics, inadequate validation, and potential bias in the reported results.^9^^,^^10^

It is important to emphasize that, at this stage, our test is not intended to replace any gold-standard diagnostic methods. Instead, it serves as a complementary tool to support clinical decision-making by enabling the selective referral of patients for further diagnostic evaluations, ultimately reducing the time to a definitive diagnosis.

Recent studies have also attempted to utilize ATR-FTIR spectroscopy for the detection of endometriosis. They primarily rely on invasive sample types (e.g., blood) and may not accurately reflect real-world healthcare clinical settings. Souza et al.^34^ in 2025, using ATR-FTIR spectroscopy of blood plasma samples from 41 endometriosis patients and 34 asymptomatic controls, found a sensitivity of 83 % and a specificity of 70 %. However, using asymptomatic controls does not simulate realistic screening conditions and may increase the diagnostic performance. Moreover, reliance on blood plasma, which is invasive and requires pre-processing, limits large-scale implementation. Kokot et al.^35^ applied ATR-FTIR spectroscopy to serum samples from 29 endometriosis patients, 24 symptomatic controls, and 18 healthy individuals. The ML algorithms' sensitivity and specificity ranged from 81 % to 100 % and 89 % to 100 %, respectively. This study primarily focused on advanced-stage endometriosis, which is easier to detect, thus limiting applicability in early or mixed-stage clinical populations.

The real challenge lies in accurately detecting endometriosis within a heterogeneous population presenting with non-specific symptoms typical of patients in secondary care facilities.^36^ In our study, the analysis of patient profile data revealed no statistically significant difference in symptoms between the positive and negative groups for endometriosis. This outcome was expected, as our study focused on a population with pelvic pain symptoms.

As demonstrated by the results, it is not feasible to differentiate groups solely based on symptoms. Patients without endometriosis often present with other gynaecological conditions that cause similar symptoms, as evidenced by the statistical differences observed in this variable. Regarding the physical examination, a difference was noted in the detection of palpable masses during the bimanual examination. However, this parameter is also non-specific. The lack of specificity in symptom and examination-based assessments further underscores the critical need for a more objective and reliable diagnostic tool.

### Limitations and future directions

While our findings demonstrate the promising potential for a ML-based urine test using ATR-FTIR spectroscopy, the sample size (*n* = 100) limits the generalizability of the algorithm’s performance. On-going patient recruitment aims to enhance the algorithm's predictive capabilities, a key focus of future studies.

Also, our cohort primarily consisted of patients with MRI-confirmed endometriosis, potentially underrepresenting early-stage cases or individuals with comorbidities that mimic endometriosis symptoms (e.g., adenomyosis, pelvic inflammatory disease). As a result, early-stage lesions, which are biochemically distinct and more challenging to detect, were not explicitly stratified in our analysis. Moreover, no formal sensitivity analysis was performed to compare model performance between early- and advanced-stage disease, which limits the statistical rigor of our findings. Future studies will prioritize a larger, multi-centre cohort that includes a more diverse population across disease stages, ethnicities, and comorbid conditions to address the limitations of AI bias and potential recruitment bias. Additionally, the authors plan to integrate MRI results with histopathological findings from laparoscopic examinations; however, achieving a statistically significant sample size for this analysis will require substantially more time.

The main limitation to integrating the ML-based urine test is the initial cost of the spectrometer (∼R$ 200,000). However, public-private partnerships could facilitate the deployment of equipment in high-demand healthcare centers. The spectral acquisition process itself is rapid and straightforward, with the algorithm available on a user-friendly graphical interface. The final report provides an easily interpretable result, indicating the probability that a patient has endometriosis. Test results would be communicated to patients through their physicians during the same consultation.

## Conclusion

Early detection of endometriosis remains a clinical challenge due to its heterogeneous and non-specific symptoms, which often overlap with other medical conditions. Our study demonstrated the potential of a Machine Learning (ML)-based urine test as a diagnostic aid for endometriosis screening. This non-invasive test utilizes Machine Learning (ML) algorithms to predict a patient's condition within 40 s of spectroscopic analysis. The sensitivity-optimized algorithm achieved a sensitivity of 93 % and a specificity of 57 %. By applying this model in clinical practice, patients with a high likelihood of endometriosis can be prioritized for pelvic MRI within one to two weeks, while unnecessary imaging referrals can be reduced, thereby aiding clinical decision-making. This test can decrease MRI utilization by approximately 42 % per 1,000 patients, resulting in significant time and cost savings, particularly in low-resource settings such as Brazil. Our study represents a preliminary validation with a limited sample size, estimating the test’s performance. Further validation will be crucial for comprehensively evaluating the algorithm’s potential.

## Funding

This work was supported by FAPES (Women in Science, #014/2022; PROFIX; UNIVERSAL); Conselho Nacional de Desenvolvimento Científico e Tecnológico (CNPq) and National Institute of Science and Technology ‒ INCT (In)Physical Activity and Health, CNPq, Brazil.

## CRediT authorship contribution statement

**Matthews Silva Martins:** Data curation, Formal analysis, Investigation, Software, Writing – original draft. **Gabriela Barros Valente:** Data curation, Writing – review & editing. **Yasmin do Nascimento Pedra:** Data curation, Writing – review & editing. **Thaís Campos Ribeiro:** Data curation, Writing – review & editing. **Neide Aparecida Tosato Boldrini:** Conceptualization, Project administration, Supervision. **Mara Rejane Barroso Barcelos:** Investigation, Data curation, Supervision, Writing – review & editing, Funding acquisition. **Francis L. Martin:** Conceptualization, Writing – review & editing. **Karin Kneipp Costa Rossi:** Data curation, Writing – review & editing. **Valerio Garrone Barauna:** Conceptualization, Funding acquisition, Investigation, Methodology, Project administration, Supervision, Writing – review & editing.

## Declaration of competing interest

The authors declare that they have no known competing financial interests or personal relationships that could have appeared to influence the work reported in this paper.

## Appendix Supplementary materials

Image, application 1
