# Supplementary material for: A machine learning approach towards endometriosis screening using infrared spectra of urine
Source: Clinics (Sao Paulo). 2025 Sep 6;80:100760. doi: 10.1016/j.clinsp.2025.100760 (PMC12450736; doi:10.1016/j.clinsp.2025.100760)

CLINICS-D-25-00093_Supplementary Material

**Table S1** Comparison of screening tools for endometriosis described in the literature.

| **Screening tools** | **Performance metrics estimates [95% CI]** | **N of women** | **Reference** |
| --- | --- | --- | --- |
| *Urinary biomarkers* |  |  |  |
| VDBP | Sensitivity 0.58 [0.44, 0.71] | 95 | Cho, 2012 |
|  | Specificity 0.55 [0.38, 0.71] |  |  |
| enolase I | Sensitivity 0.56 [0.40, 0.72] | 59 | Yun, 2014 |
|  | Specificity 0.70 [0.46, 0.88] |  |  |
| CK 19 | Sensitivity: 0.11 [0.05, 0.22] | 98 | Lessey, 2014 |
|  | Specificity: 0.94 [0.81, 0.99] |  |  |
| peptide m/z 1824.3 Da | Sensitivity: 0.77 [0.46, 0.95] | 28 | El-Kasti, 2011 |
|  | Specificity: 0.73 [0.45, 0.92] |  |  |
| peptide m/z 1767.1 Da | Sensitivity: 0.75 [0.43, 0.95] | 27 | El-Kasti, 2011 |
|  | Specificity: 0.87 [0.60, 0.98] |  |  |
| peptide m/z 2052.3 Da | Sensitivity: 0.83 [0.71, 0.92] | 122 | Wang, 2014 |
|  | Specificity: 0.69 [0.56, 0.80] |  |  |
| peptide m/z 3393.9 Da | Sensitivity: 0.85 [0.73, 0.93] | 122 | Wang, 2014 |
|  | Specificity: 0.71 [0.58, 0.82] |  |  |
| peptide m/z 1579.2 Da | Sensitivity: 0.83 [0.71, 0.92] | 122 | Wang, 2014 |
|  | Specificity: 0.69 [0.56, 0.80] |  |  |
| peptide m/z 891.6 Da | Sensitivity: 0.82 [0.70, 0.90] | 122 | Wang, 2014 |
|  | Specificity: 0.65 [0.51, 0.76] |  |  |
| 5 peptides m/z 1433.9 + 1599.4 + 2085.6 + 6798.0 + 3217.2 Da | Sensitivity: 0.91 [0.59, 1.00] | 25 | Wang, 2014 |
|  | Specificity: 0.93 [0.66, 1.00] |  |  |
| *Blood biomarkers* |  |  |  |
| Glycodelin-A | Sensitivity: 0.82 [0.70, 0.91] | 99 | Kocbek, 2013 |
|  | Specificity: 0.79 [0.63, 0.90] |  |  |
| IGFBP-3 | Sensitivity: 0.55 [0.42, 0.68] | 99 | Vodolazkaia, 2012 |
|  | Specificity: 0.44 [0.28, 0.60] |  |  |
| VEGF | Sensitivity: 0.50 [0.37, 0.63] | 99 | Vodolazkaia, 2012 |
|  | Specificity: 0.61 [0.45, 0.76] |  |  |
| Urocortin | Sensitivity: 0.76 [0.61, 0.88] | 88 | Tokmak, 2011 |
|  | Specificity: 0.46 [0.31, 0.61] |  |  |
| Prolactin | Sensitivity: 0.44 [0.32, 0.58] | 97 | Bilibio, 2014 |
|  | Specificity: 0.94 [0.80, 0.99] |  |  |
| TNF-α | Sensitivity: 0.79 [0.69, 0.88] | 116 | Mihalyi, 2010 |
|  | Specificity: 0.74 [0.57, 0.87] |  |  |
| anti-endometrial antibodies | Sensitivity: 0.81 [0.76, 0.87] | 759 | Meta-analysis of multiple studies: Nisemblat, 2016 |
|  | Specificity: 0.75 [0.46, 1.00] |  |  |
| IL-6 | Sensitivity: 0.63 [0.52, 0.75]  309 | 309 | Meta-analysis of multiple studies: Nisemblat, 2016 |
|  | Specificity: 0.69 [0.57, 0.82] |  |  |
| CAE-125 | Sensitivity: 0.40 [0.32, 0.49] | 775 | Meta-analysis of multiple studies: Nisemblat, 2016 |
|  | Specificity: 0.91 [0.88, 0.94] |  |  |
| *Symptom-based tools* |  |  |  |
| Physical and demographic characteristics, family history, symptoms, and quality of life (5 variables) | Sensitivity = 0.80 | 90 | Yeung, 2014 |
|  | Specificity = 0.57 |  |  |
| Endometriosis Index based on patient pain evaluation and physician consultation (38 variables) | Sensitivity = 0.72 | 120 | Fasciani, 2010 |
|  | Specificity = 0.90 |  |  |
|  | ^a^For deep infiltrating endometriosis |  |  |
| Presurgical diagnosis endometriosis using a 7-item questionnaire | Specificity = 0.88 | 157 | Fedele, 2007 |
|  | Specificity = 0.88 |  |  |
|  | ^a^For bladder endometriosis |  |  |
| 56 Endometriosis symptoms collected via an online questionnaire | Sensitivity = 0.93 | 886 | Goldstein, 2023 |
|  | Sensitivity = 0.93 |  |  |
|  | ^a^Self-reported diagnosis |  |  |

^a^ Confidence intervals for urinary biomarkers and blood biomarkers are provided by Liu et al. 2015 and Nisenblat et al. 2016, respectively.

**References**

Liu E, Nisenblat V, Farquhar C, Fraser I, Bossuyt PMM, Johnson N, et al. Urinary biomarkers for the non‐invasive diagnosis of endometriosis. Cochrane Database Syst Rev. 2015;2015(12):CD012019.

Cho S, Choi YS, Yim SY, Yang HI, Jeon YE, Lee KE, et al. Urinary vitamin D- binding protein is elevated in patients with endometriosis. Hum Reprod. 2012;27(2):515-22.

Yun BH, Lee YS, Chon SJ, Jung YS, Yim SY, Kim HY, et al. Evaluation of elevated urinary enolase I levels in patients with endometriosis. Biomarkers 2014;19(1):16-21.

Lessey BA, Savaris RF, Ali S, Brophy S, Tomazic-Allen S, Chwalisz K. Diagnostic Accuracy of Urinary Cytokeratin 19 Fragment for Endometriosis. Reprod Sci. 2015;22(5):551-5.

El-Kasti MM, Wright C, Fye HK, Roseman F, Kessler BM, Becker CM. Urinary peptide profiling identifies a panel of putative biomarkers for diagnosing and staging endometriosis. Fertil Steril. 2011;95(4):1261-6.e1-6..

Wang L, Liu HY, Shi HH, Lang JH, Sun W. Urine peptide patterns for non-invasive diagnosis of endometriosis:a preliminary prospective study. Eur J Obstet Gynecol Reprod Biol. 2014;177:23-8.

Nisenblat V, Bossuyt PMM, Shaikh R, Farquhar C, Jordan V, Scheffers CS, et al. Blood biomarkers for the non‐invasive diagnosis of endometriosis. Cochrane Database Syst Rev. 2016;2016(5):CD012179.

Kocbek V, Vouk K, Mueller MD, Rizner TL, Bersinger NA. Elevated glycodelin-A concentrations in serum and peritoneal fluid of women with ovarian endometriosis. Gynecol Endocrinol. 2013;29(5):455-9.

Vodolazkaia A, El-Aalamat Y, Popovic D, Mihalyi A, Bossuyt X, Kyama CM, et al. Evaluation of a panel of 28 biomarkers for the non-invasive diagnosis of endometriosis. Hum Reprod. 2012;27(9):2698-711.

Tokmak A, Ugur M, Tonguc E, Var T, Moraloglu O, Ozaksit G. The value of urocortin and Ca-125 in the diagnosis of endometrioma. Arch Gynecol Obstet. 2011;283(5):1075-9.

Bilibio JP, Souza CA, Rodini GP, Andreoli CG, Genro VK, de Conto E, et al. Serum prolactin and CA-125 levels as biomarkers of peritoneal endometriosis. Gynecol Obstet Invest. 2014;78(1):45-52.

Mihalyi A, Gevaert O, Kyama CM, Simsa P, Pochet N, De Smet F, et al. Non- invasive diagnosis of endometriosis based on a combined analysis of six plasma biomarkers. Hum Reprod. 2010;25(3):654-64.

Yeung JR, Patrick; Bazinet, Caroline; Gavard, Jeffrey A. Development of a symptom-based, screening tool for early-stage endometriosis in patients with chronic pelvic pain. J Endometriosis Pelvic Pain Disorders. 2014;6(4):174-89.

Fasciani A, Repetti F, Binda GA, Puntoni M, Meroni MG, Bocci G. Endometriosis index: a software-derived score to predict the presence and severity of the disease. J Endometriosis. 2010;2(2):79-86.

Fedele L, Bianchi S, Carmignani L, Berlanda N, Fontana E, Frontino G. Evaluation of a new questionnaire for the presurgical diagnosis of bladder endometriosis. Hum Reprod. 2007;22(10):2698-701.

Surrey E, Carter CM, Soliman AM, Khan S, DiBenedetti DB, Snabes MC, et al. Patient-completed or symptom-based screening tools for endometriosis: a scoping review. Arch Gynecol Obstet. 2017;296(2):153-65.

Goldstein A, Cohen S. Self-report symptom-based endometriosis prediction using machine learning. Sci Rep. 2023;13(1):5499.

**Figure S1** Raw spectra of urine samples from 100 patients, each analyzed in triplicate.


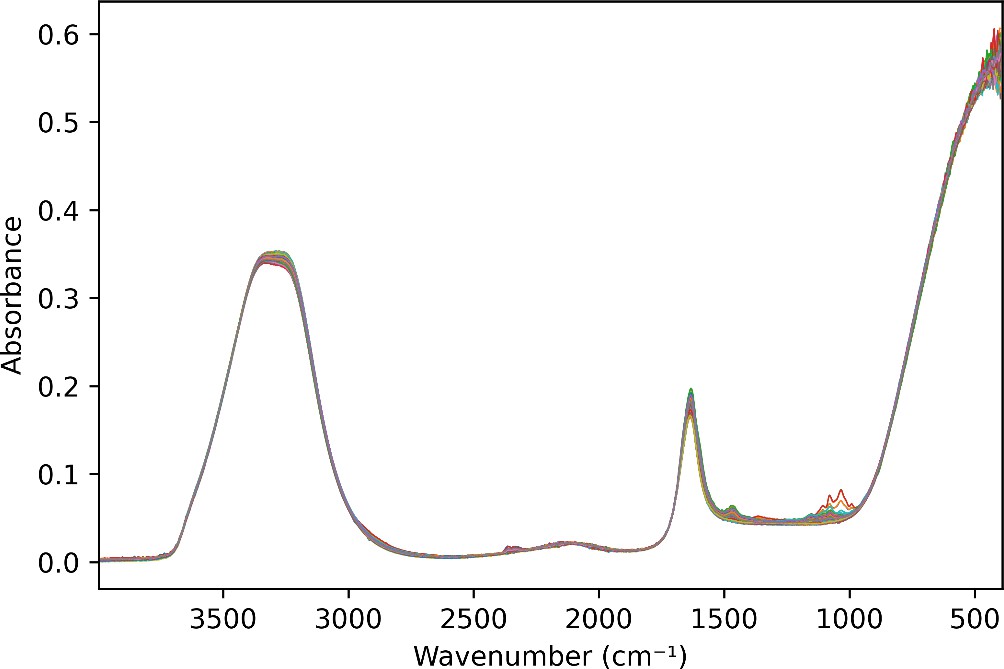


**Figure S2** Averaged and pre-processed spectra of urine samples from 100 patients.


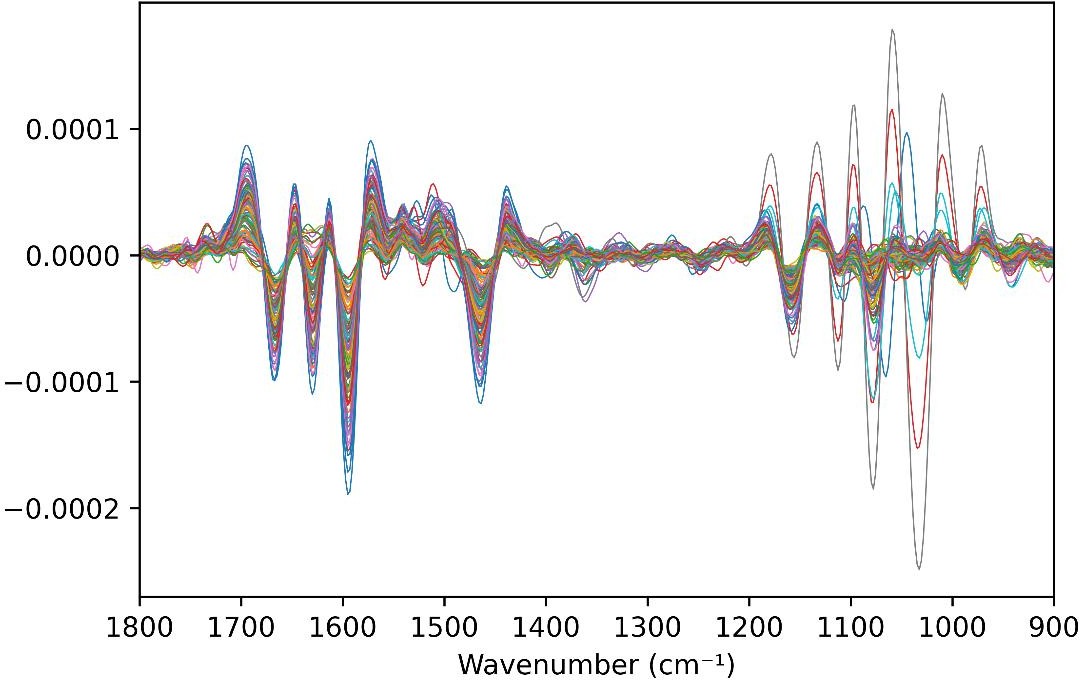


**Figure S3** Frequency of variables selected by the 100 models during the algorithm’s training. The variable with the highest selection frequency is normalized to 100%.


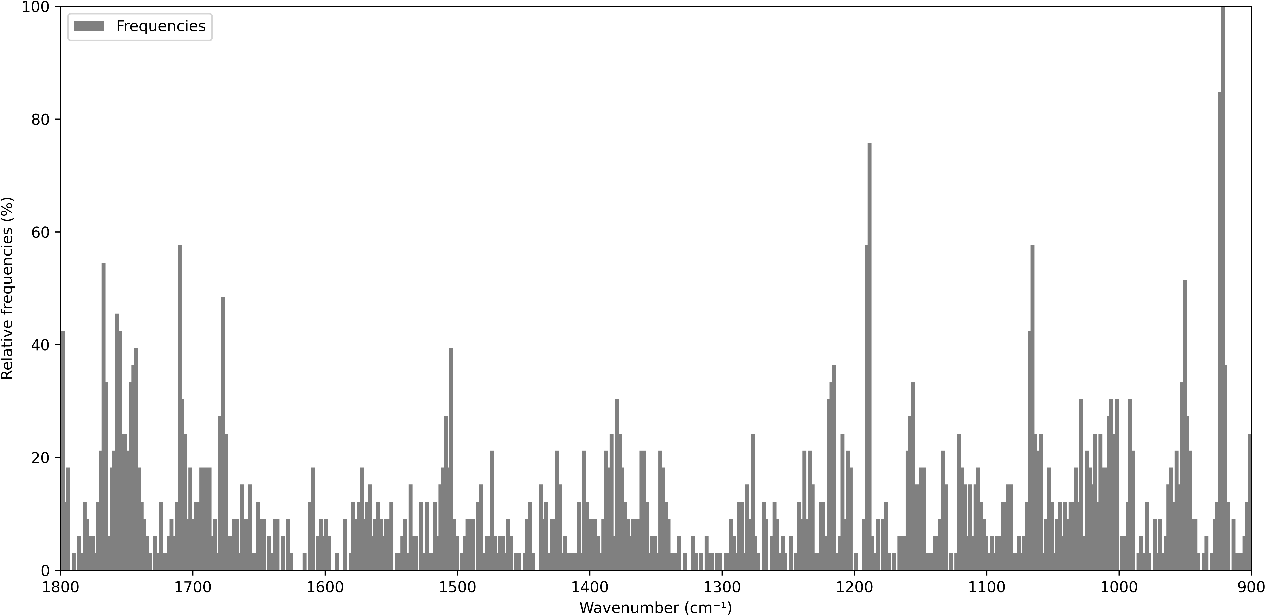

Supplement: Supplementary file 1 [file mmc1.docx]
